# Mapping and visualization of global research progress on autophagy in metabolic dysfunction-associated steatotic liver disease and metabolic syndrome: a bibliometric analysis (2009–2024)

**DOI:** 10.3389/fmed.2025.1525526

**Published:** 2025-06-25

**Authors:** Xing Liu, Xiang Li

**Affiliations:** ^1^Shenzhen Pingle Orthopedic Hospital (Shenzhen Pingshan Traditional Chinese Medicine Hospital), Shenzhen, Guangdong, China; ^2^School of Public Health (Shenzhen), Sun Yat-sen University, Shenzhen, Guangdong, China

**Keywords:** autophagy, non-alcoholic fatty liver disease, metabolic syndrome, bibliometric analysis, VOSviewer, CiteSpace

## Abstract

**Objective:**

This study aimed to delineate the current trends and hotspots in autophagy research related to metabolic syndrome (MetS) and metabolic dysfunction-associated steatotic liver disease (MASLD), with the aim of guiding future investigations in this area.

**Methods:**

This study extracted research on autophagy in MetS and MASLD from the Web of Science Core Collection (WoSCC) database. Review articles were systematically excluded to focus on original research contributions. A bibliometric analysis and visualization were conducted using VOSviewer 1.6.20, CiteSpace 6.3.R1, and R 4.3.3.

**Results:**

The study included 1,114 articles from 1,220 institutions across 42 countries/regions, demonstrating a significant increase in research output from 2009 to 2024. China led with 506 publications, followed by the USA and Korea. The Egyptian Knowledge Bank constitutes a consortium of institutions operating within the national research framework, with one institution designated as the primary publishing entity. Notably, the journal Nature has emerged as the most frequently cited publication. Singh Rajat received the highest number of citations (3,610), while Marycz Krzysztof was the most prolific author. The most cited article, published in 2009, was titled “Autophagy regulates lipid metabolism.” Keyword trends have shifted from earlier topics such as “phosphorylation” and “gene-expression” to more recent terms like “lipid accumulation” and “mitophagy.” Burst keyword analysis indicated that “liver fibrosis,” “modulation,” “gut microbiota,” and “lipotoxicity” have emerged as significant topics.

**Conclusions:**

This study has elucidated the protective role of autophagy in MASLD and MetS. Future research is anticipated to concentrate on the activation of autophagy in the context of natural product drug discovery, the exploration of underlying molecular mechanisms, the regulation of fatty acid metabolism, and the development of functional nutritional supplements, among other relevant areas.

## 1 Introduction

Metabolic dysfunction-associated steatotic liver disease (MASLD) currently affects ~30% of the global population, translating to an estimated 314 million individuals (1–4). As the hepatic manifestation of metabolic syndrome (MetS) (5–7), MASLD is intricately linked to insulin resistance, obesity, and dyslipidemia, thereby representing a substantial public health concern (8–10). The disease spectrum ranges from simple steatosis to non-alcoholic steatohepatitis (NASH), fibrosis, and hepatocellular carcinoma, all of which are influenced by factors such as dysregulated lipid metabolism, oxidative stress, inflammation, and mitochondrial dysfunction (9, 10).

Autophagy is a highly conserved cellular degradation process that is pivotal in mitigating the progression of MASLD by facilitating the clearance of lipid droplets, a process referred to as “macrolipophagy,” as well as damaged organelles. Furthermore, autophagy plays a critical role in suppressing oxidative stress and inflammation (11–13). Compromised autophagic flux in MASLD is significantly associated with increased hepatocyte apoptosis, thereby exacerbating the severity of the condition (14). Dysregulation of autophagy exacerbates hepatic lipid accumulation and insulin resistance, thereby contributing to complications associated with MetS (13). Recent advancements underscore the significance of autophagy modulation as a viable therapeutic strategy. Natural products, including polyphenols and saponins, along with pharmacological agents, target key pathways such as AMPK/mTOR, TFEB-mediated lysosomal biogenesis, and PINK1/Parkin-dependent mitophagy (15–21). For instance, quercetin has been shown to activate AMPK, thereby enhancing mitochondrial autophagy, while resveratrol mitigates endoplasmic reticulum (ER) stress via SIRT1 signaling (15–18). Moreover, TFEB agonists, such as formononetin, promote lipophagy, thereby providing valuable insights into the restoration of lipid homeostasis (19–21). Importantly, alongside natural and pharmacological compounds, clinical interventions aimed at promoting weight loss—such as dietary modifications and bariatric surgery—have demonstrated a significant capacity to enhance hepatic autophagic flux in individuals with MASLD (22, 23). Employing mathematical and statistical methodologies, bibliometric analysis yields insights into publication trends, predominant research domains, co-authorship dynamics, keyword frequency, and citation patterns over time. This approach facilitates a comprehensive overview of the academic landscape pertaining to autophagy in MASLD and MetS (24).

Bibliometric analyses elucidate the evolving research trends that underscore the significance of autophagy in MASLD and MetS, while concurrently identifying emerging therapeutic candidates. These investigations reveal an increasing interest in compounds such as berberine (which targets the SIRT1/AMPK axis), vitamin D (which promotes fatty acid β-oxidation), and probiotics (which modulate the gut-liver axis) (25–27). Nevertheless, substantial gaps persist in our comprehension of tissue-specific autophagy regulation and the translational potential of preclinical findings into clinical applications. This review synthesizes 15 years of research to delineate the landscape of MASLD/MetS and autophagy, highlighting pivotal discoveries, unresolved inquiries, and therapeutic innovations. By integrating mechanistic insights with bibliometric trends, we aim to steer future research toward targeted interventions for metabolic liver diseases.

## 2 Materials and methods

### 2.1 Data sources and search strategy

The literature search was conducted to retrieve relevant articles from the inception of the database until October 2024 from the Web of Science Core Collection (WoSCC) (28). The search strategy is delineated in Supplementary Table 1. This study included exclusively “articles” and considered only documents written in English. Given that all data were obtained from a publicly accessible database, ethical declarations or approvals are not necessary.

### 2.2 Data analysis and visualization

We extracted relevant data from the retrieved article titles and used Microsoft Excel 365 to identify and calculate bibliometric parameters. These metrics cover key aspects of articles, including the number of publications per year, citation frequency, average citation fre-quency, journal title, journal impact factor, country/region of article, publishing institutions, and authors. The visualization and analysis process involved the use of three powerful bibliometric analysis tools to fully analyze the academic data: VOSviewer 1.6.20, CiteSpace 6.3.R1, and R bibliometrix package 4.3.3. VOSviewer is a multifaceted software tool that is instrumental in mapping institutional collaborations, co-authorships, citations, and co-citations (29). It was employed to perform keyword co-occurrence analysis. CiteSpace 6.3.R1 was used for country, institution, and author, the intermediary centrality of keyword emergence detection and co-occurrence analysis (30), with the parameters set to time slicing: from January 2009 to October 2024 (research in this field was originally published in 2009). Network pruning techniques, including pathfinder and clip merge, enhanced the visualizations and improved their interpretability. This refinement enabled the identification of significant shifts in research focus and underscored emerging areas of interest in autophagy related to MetS and MASLD research. Furthermore, the R-based bibliometric package “bibliometrix” was employed for a thorough evaluation of research output, global distributions, and the performance metrics of authors and journals (31). Metrics such as the h-index, g-index, and m-index were calculated to assess the academic impact of authors and institutions (32, 33). Journal performance was evaluated utilizing the most recent 2024 data from Journal Citation Reports (JCR) quartiles and Impact Factors (IF), thereby ensuring timely assessments. These metrics provided critical insights into the academic influence and prestige of journals within the field. By integrating these analytical methodologies, this study presents a systematic overview of research pertaining to autophagy in MetS and MASLD, emphasizing significant contributors, leading institutions, and emerging research trends. The findings offer invaluable guidance for future investigations and promote collaborative opportunities aimed at enhancing the understanding and treatment of MetS and MASLD.

## 3 Results

### 3.1 Overview of main findings

**Figure 1:**
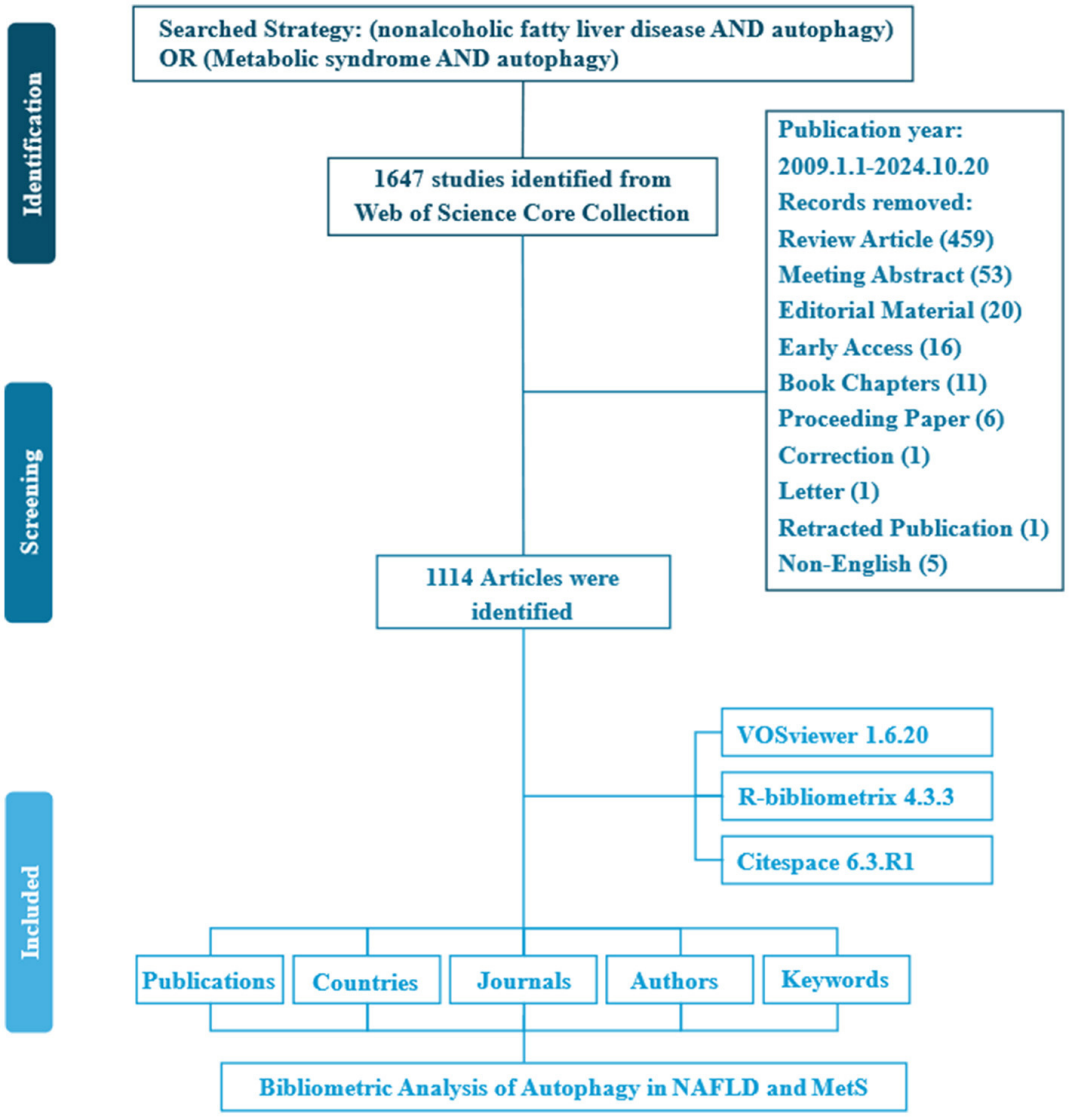
Flowchart of the literature screening process.

**Figure 2:**
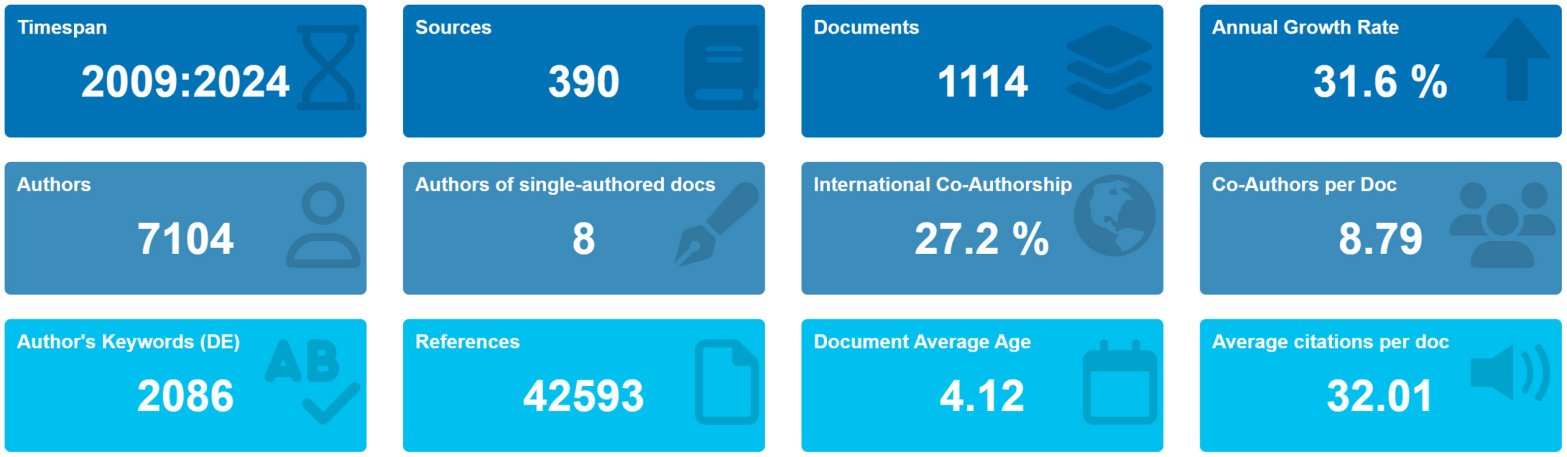
Overview of the main information.

A total of 1,647 studies were retrieved from the WoSCC database. To concentrate on original research contributions, review articles (designated as “review” in WoSCC) were systematically excluded. Consequently, 459 review articles were removed during the screening process. The study flowchart is illustrated in Figure 1. Over the past 15 years, a total of 1,114 articles were contributed by 7,104 authors from 1,220 institutions across 42 countries, and these articles appeared in 390 distinct journals. Collectively, these publications cited 42,593 references, demonstrating a robust engagement with the existing body of literature. The average age of the documents was 4.12 years, with each article receiving an average of 32.01 citations, underscoring the enduring relevance and impact of research in this field (Figure 2).

### 3.2 Annual publication trends

**Figure 3:**
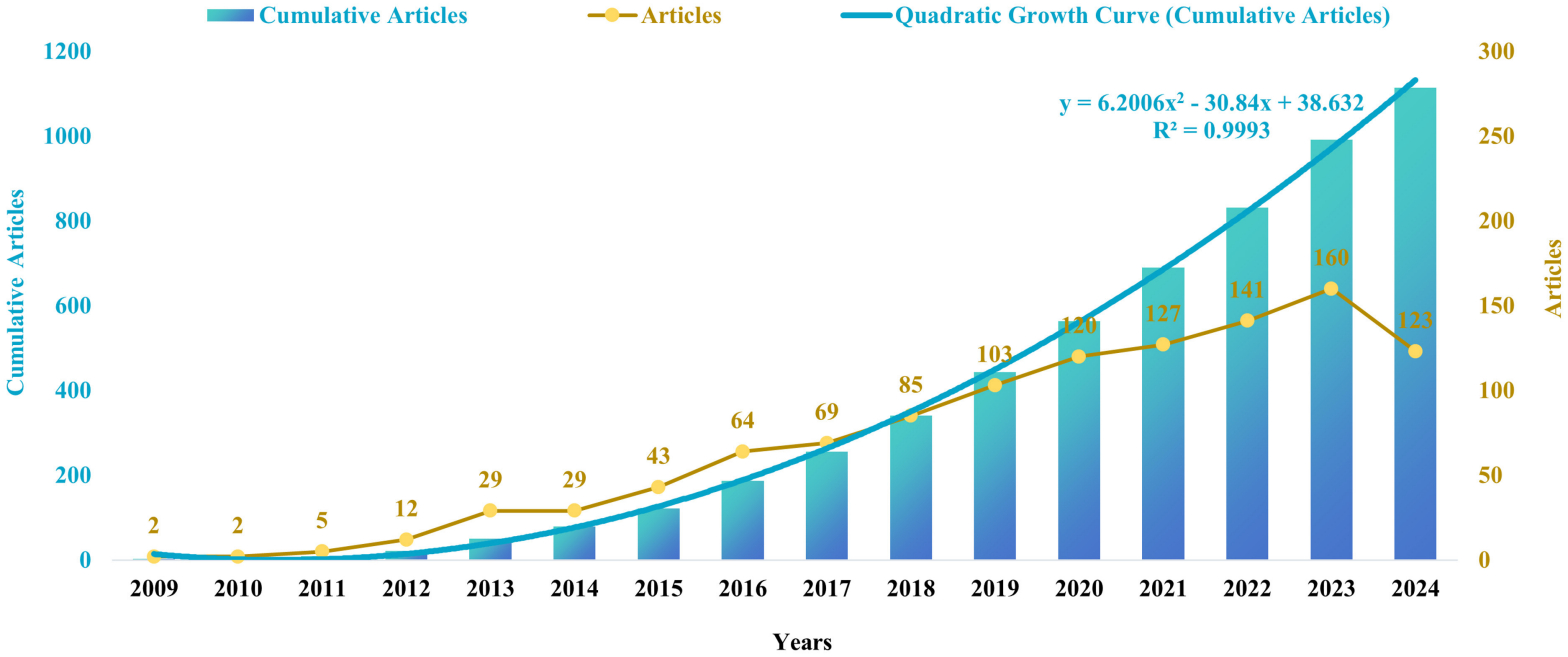
Annual numbers of publications on autophagy in NAFLD and MetS.

To gain insight into the evolution of research in this field, we conducted an analysis of annual publication trends. Our study period indicated a pronounced upward trajectory in both annual and cumulative publications, particularly since 2010. The increase in cumulative pub-lications over time is consistent with the quadratic growth equation *y* = 6.2006 *x*^2^-30.84 *x*+38.632, which exhibits a coefficient of determination (*R*^2^) of 0.9993 and an annual growth rate of 31.60%. Notably, the year 2023 recorded the highest number of publications, accounting for 14.36% of the total (Figures 2, 3).

### 3.3 Analysis of countries

**Figure 4:**
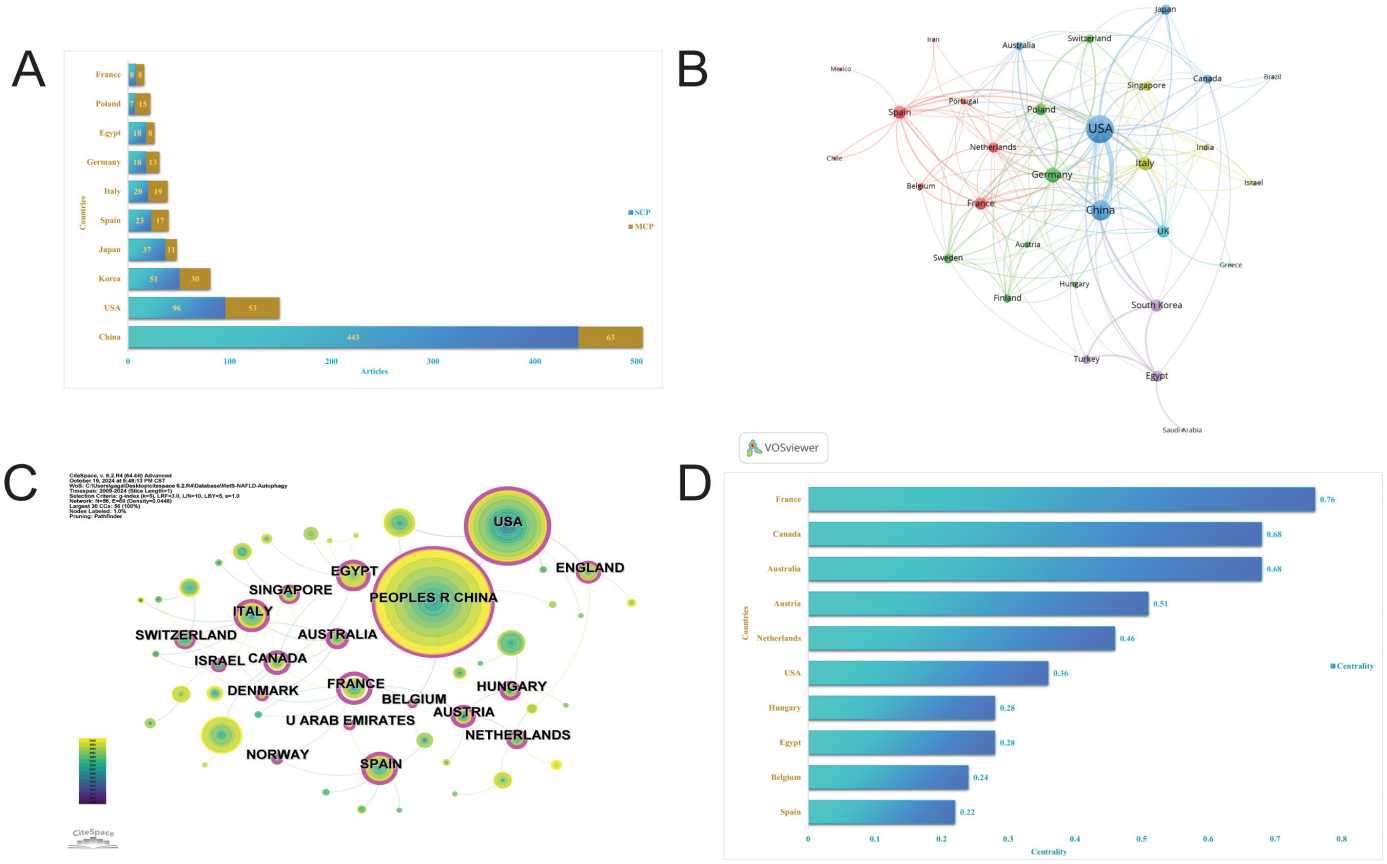
Visualization of countries. **(A)** Articles by countries. **(B)** International collaboration network of countries by VOS viewer. **(C)** International collaboration network of countries by CiteSpace. **(D)** Intermediary centrality of countries.

The identified publications originated from 42 countries, with China leading with 506 articles, accounting for 45.40% of all articles. Other significant contributors included the USA (149 articles), Korea (81 articles), Japan (48 articles), Spain (40 articles), and Italy (39 articles; Figure 4A, Table 1). Despite China's dominance in total publication volume, the USA, Canada, and Singapore exhibited the highest average citation rates, with averages of 69.80, 53.50, and 47.90, respectively. Seven countries demonstrated high betweenness centrality (>0.1), ranked in descending order as India, Australia, the USA, Italy, Spain, England, and Brazil (Figures 4C, D). Furthermore, country collaborations were visualized using VOSviewer, indicating that the USA, China, and Italy constituted the most robust international collaboration network (Figure 4B).

**Table 1:** Publication and citation profiles of leading countries.

**Country**	**Articles**	**Freq**	**MCP %**	**TC**	**TC_rank**	**TP**	**TP_rank**	**Average citations**
China	506	0.454	12.5	11,898	1	1,823	1	23.50
USA	149	0.134	35.6	10,405	2	727	2	69.80
Korea	81	0.073	37.0	2,251	3	341	3	27.80
Japan	48	0.043	22.9	1,151	6	175	7	24.00
Spain	40	0.036	42.5	1,592	5	269	4	39.80
Italy	39	0.035	48.7	1,831	4	200	5	46.90
Germany	31	0.028	41.9	675	7	187	6	21.80
Egypt	26	0.023	30.8	261	14	103	9	10.00
Poland	22	0.020	68.2	597	9	66	11	27.10
France	16	0.014	50.0	504	12	117	8	31.50

Articles: Publications of corresponding authors only; Freq: frequence of total publications; MCP %: proportion of multiple country publications; TP: total publications; TP_rank: rank of total publications; TC: total citations; TC_rank: rank of total citations; Average citations: the average number of citations per publication.

### 3.4 Analysis of institutions

**Figure 5:**
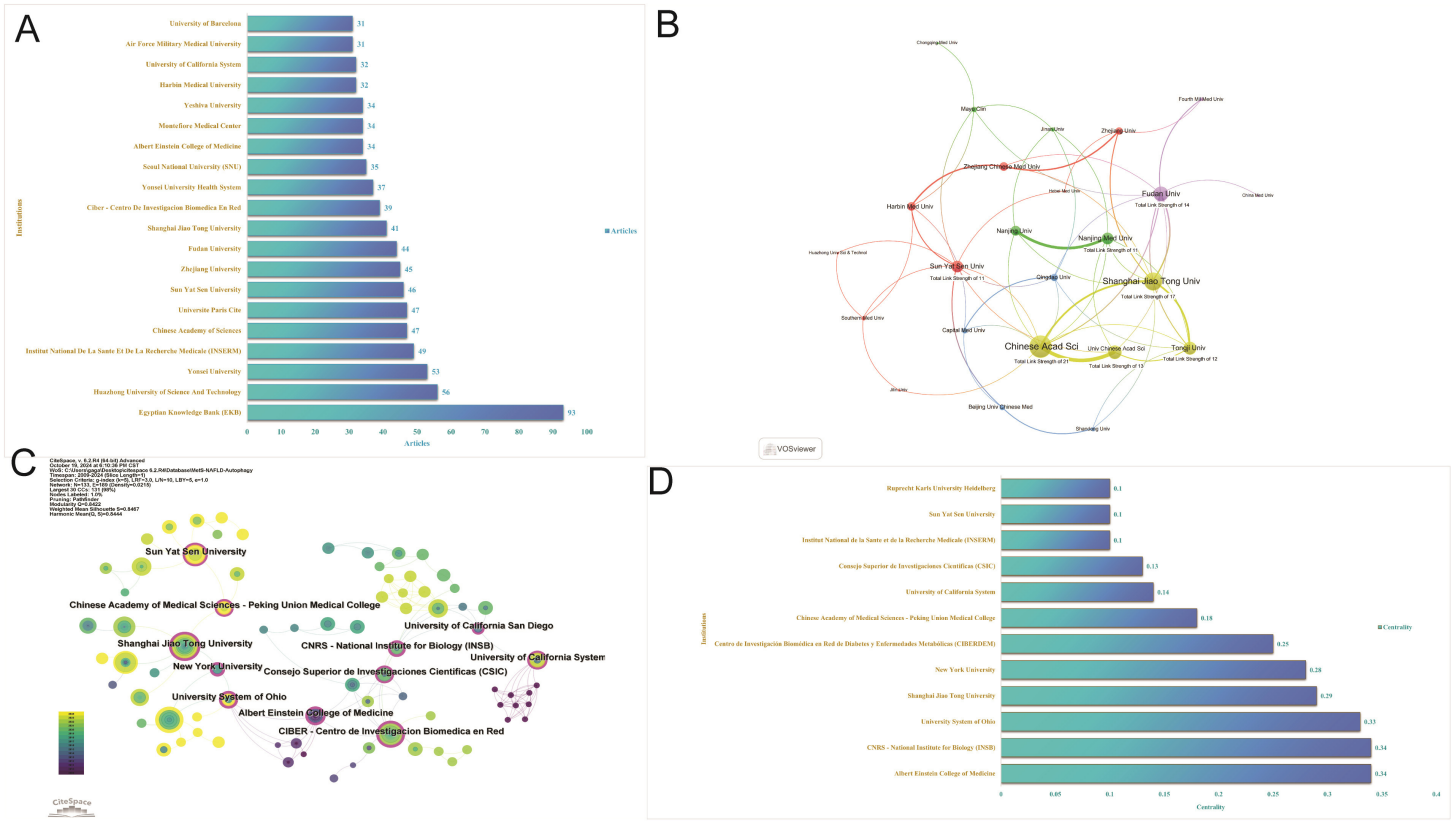
Visualization of institutions. **(A)** Articles of institutions. **(B)** Collaborative networks of institutions by VOS viewer. **(C)** Collaborative networks of institutions by CiteSpace. **(D)** Intermediary centrality of institutions.

Research publications on autophagy in MASLD and MetS involved a total of 1,220 institutions. The institutions with the highest number of publications were the Egypt Knowledge Bank (Egypt, 93 articles), HuaZhong University of Science and Technology (China, 56 articles), and Yonsei University (South Korea, 53 articles; Figure 5A). The analysis of collaborative networks focused on institutions with a minimum of 10 articles and was visualized using VOSviewer. Clusters were color-coded to reflect the frequency of collaboration among institutions (Figure 5B). Notably, the Chinese Academy of Sciences exhibited the largest node, indicating the highest level of collaboration with other institutions. Furthermore, Figures 5C, D illustrate the significance of institutions operating as intermediaries. The Albert Einstein College of Medicine possesses a betweenness centrality score of 0.34, positioning it as a vital hub for cross-regional collaboration. This status is further augmented by the Centro de Investigación Biomecánica en Red de Diabetes y Enfermedades Metabólicas, which has a betweenness centrality score of 0.25.

### 3.5 Analysis of journals

**Figure 6:**
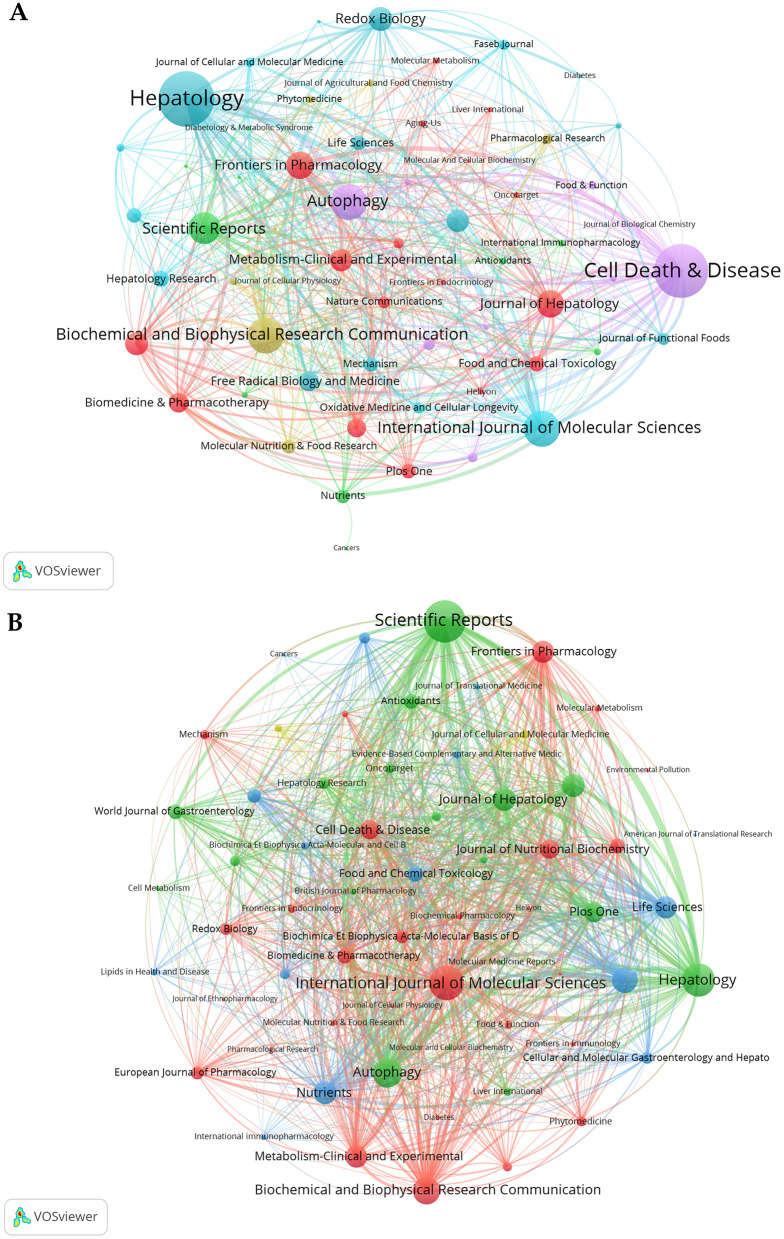
Co-occurrence and bibliographic coupling analysis. **(A)** The co-occurrence networks of journals. **(B)** The bibliographic coupling networks of journals.

Research on autophagy in MASLD and MetS is significantly represented across 390 academic journals. *Scientific Reports* leads this body of work with 36 articles, which constitutes 3.23% of the overall total. This is succeeded by the *International Journal of Molecular Sciences* and *Biochemical and Biophysical Research Communications*, which have published 28 and 26 articles, respectively, representing 2.51 and 2.33% of the total (Table 2). Co-citation analysis has identified the five key journals with the highest total link strength as follows: *Metabolism: Hepatology* (118), *Cell Death & Disease* (114), *Autophagy* (77), *International Journal of Molecular Sciences* (77), and *Biochemical and Biophysical Research Communications* (76) (Figure 6A). Similarly, bibliographic coupling analysis has revealed the five key journals with the highest total link strength: *Scientific Reports* (7,068), *International Journal of Molecular Sciences* (5,931), *Hepatology* (5,535), *Autophagy* (4,912), and *Biochemical and Biophysical Research Communications* (4,691; Figure 6B).

**Table 2:** Bibliometric indicators of high-impact journals.

**Source**	**h_index**	**JCR**	**IF_2024**	**TC**	**TC_rank**	**TP**	**TP_rank**	**PY_start**
Scientific reports	21	1	3.8	1,118	4	36	1	2014
Hepatology	17	1	12.9	1,527	2	21	4	2013
Autophagy	15	1	14.6	1,045	6	17	7	2016
Plos one	14	1	2.9	1,282	3	18	5	2011
International Journal of Molecular Sciences	13	1	5.6	737	12	28	2	2014
Biochemical and biophysical research communications	13	2	3.1	440	16	26	3	2013
Journal of Hepatology	13	2	26.8	811	9	17	8	2014
Cell death & disease	12	1	8.1	1,006	7	15	10	2014
Redox biology	11	1	10.6	840	8	12	15	2013
Free radical biology and medicine	10	1	7.1	553	13	15	11	2013

H_index: the h-index of the journal, which measures both the productivity and citation impact of the publications; IF: impact factor, indicating the average number of citations to recent articles published in the journal; JCR_quartile: The quartile ranking of the journal in the Journal Citation Reports, indicating the journal's ranking relative to others in the same field (Q1: top 25%, Q2: 25%−50%, Q3: 50%−75%, Q4: bottom 25%); TP: total publications; TP_rank: rank of total publications; TC: total citations; TC_rank: rank of total citations; Average citations: the average number of citations per publication; PY_start: publication year start, indicating the year the journal started publication.

### 3.6 Analysis of authors and collaborations

**Figure 7:**
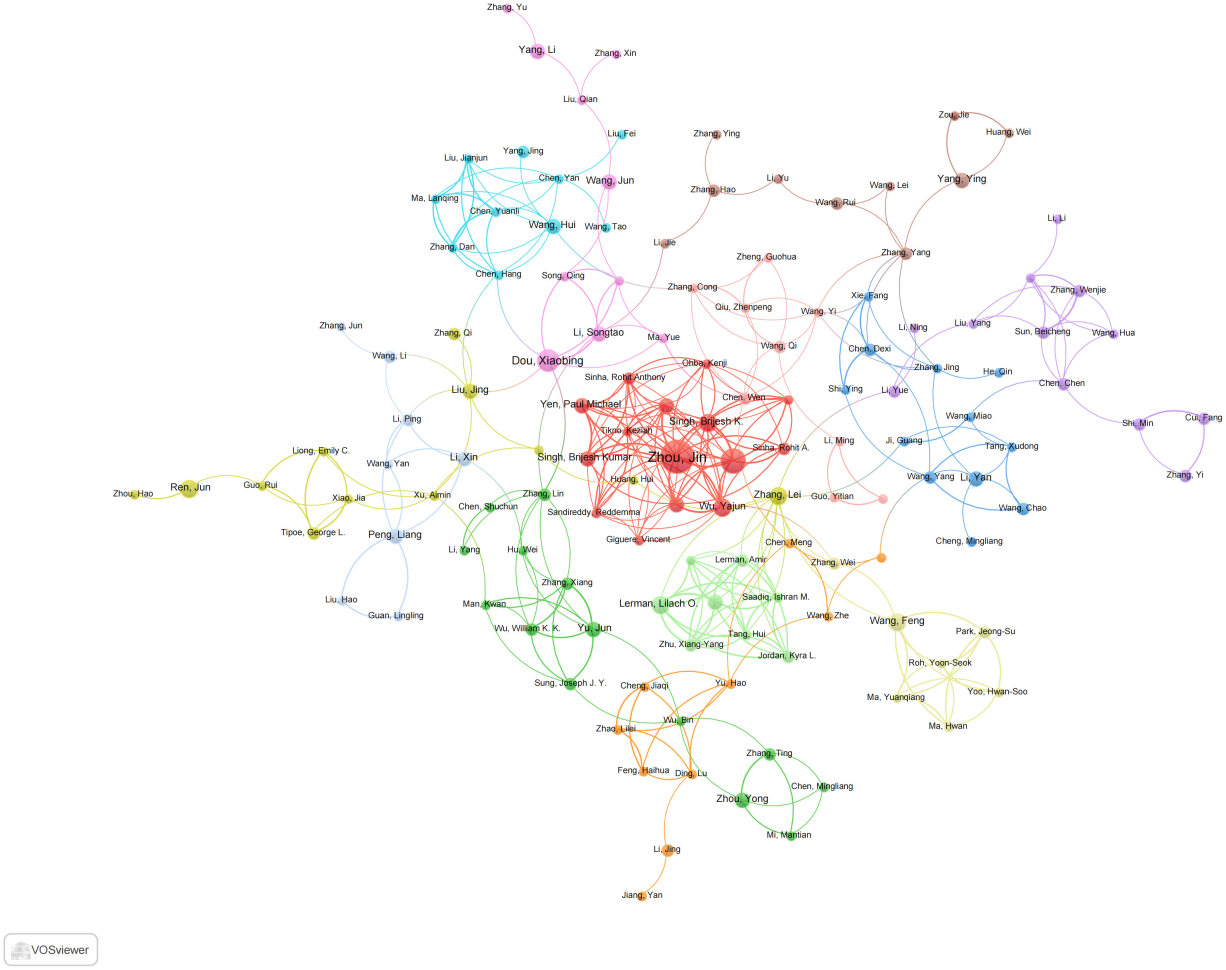
Visualization map depicting the collaboration among different authors.

A total of 1,114 articles were authored by 8,134 contributors, indicating a broad distribution of authorship and moderate levels of scholarly collaboration. As illustrated in Table 3, authors are ranked according to their h-index (top five) and total citations (bottom five in reverse order). Krzysztof Marycz is identified as the most impactful contributor, with an h-index of 12, closely followed by Jin Zhou, who possesses an h-index of 10. Additionally, Zhou Jin exhibited the highest degree of collaborative engagement in the network analysis, achieving a total link strength of 52, surpassing Yen Paul M. (33) and Singh Brijesh K./Wu Yajun (31) (Figure 7). In terms of citation leadership, Singh Rajat, Kaushik Susmita, Ana María Cuervo, Komatsu Masaaki, and Czaja Mark J. are particularly notable, primarily due to their seminal 2009 *Nature* paper, “Autophagy regulates lipid metabolism,” which has been cited 3,003 times as of November 2024 and has significantly influenced this research domain. This contrast between productivity metrics (h-index) and citation impact highlights the enduring significance of foundational studies in comparison to contemporary research output.

**Table 3:** Publication and citation profiles of high-impact authors.

**Author**	**h_index**	**g_index**	**m_index**	**PY_start**	**TP**	**TP_rank**	**TP_frac**	**TC**	**TC_rank**
Marycz Krzysztof	12	19	1.333	2016	19	1	4.04	468	33
Zhou Jin	10	11	0.909	2014	11	2	1.04	544	29
Kornicka Katarzyna	9	10	1	2016	10	4	2.13	389	63
Yen Paul M.	7	8	0.636	2014	8	7	0.84	469	32
Jung Tae Woo	7	11	0.7	2015	11	3	1.65	177	256
Komatsu Masaaki	6	6	0.375	2009	6	15	0.45	3,200	5
Czaja Mark J.	4	5	0.235	2009	5	43	1.44	3,272	4
Cuervo Ana Maria	2	2	0.118	2009	2	405	0.24	3,278	3
Kaushik Susmita	5	5	0.176	2009	3	170	0.35	3,431	2
Singh Rajat	5	5	0.294	2009	5	36	0.48	3,610	1

H_index: the H-index of the author, which measures both the productivity and citation impact of the authors; G_index: the G-index of the author, which gives more weight to highly-cited authors; M_index: the m-index of the author, which was the H-index divided by the number of years since the first published article; TP: total publications; TP_rank: rank of total publications; TC: total citations; TC_rank: rank of total citations; Average citations: the average number of citations per author; PY_start: publication year start, indicating the year the author started publication.

### 3.7 Analysis of research hotspots and frontiers

**Figure 8:**
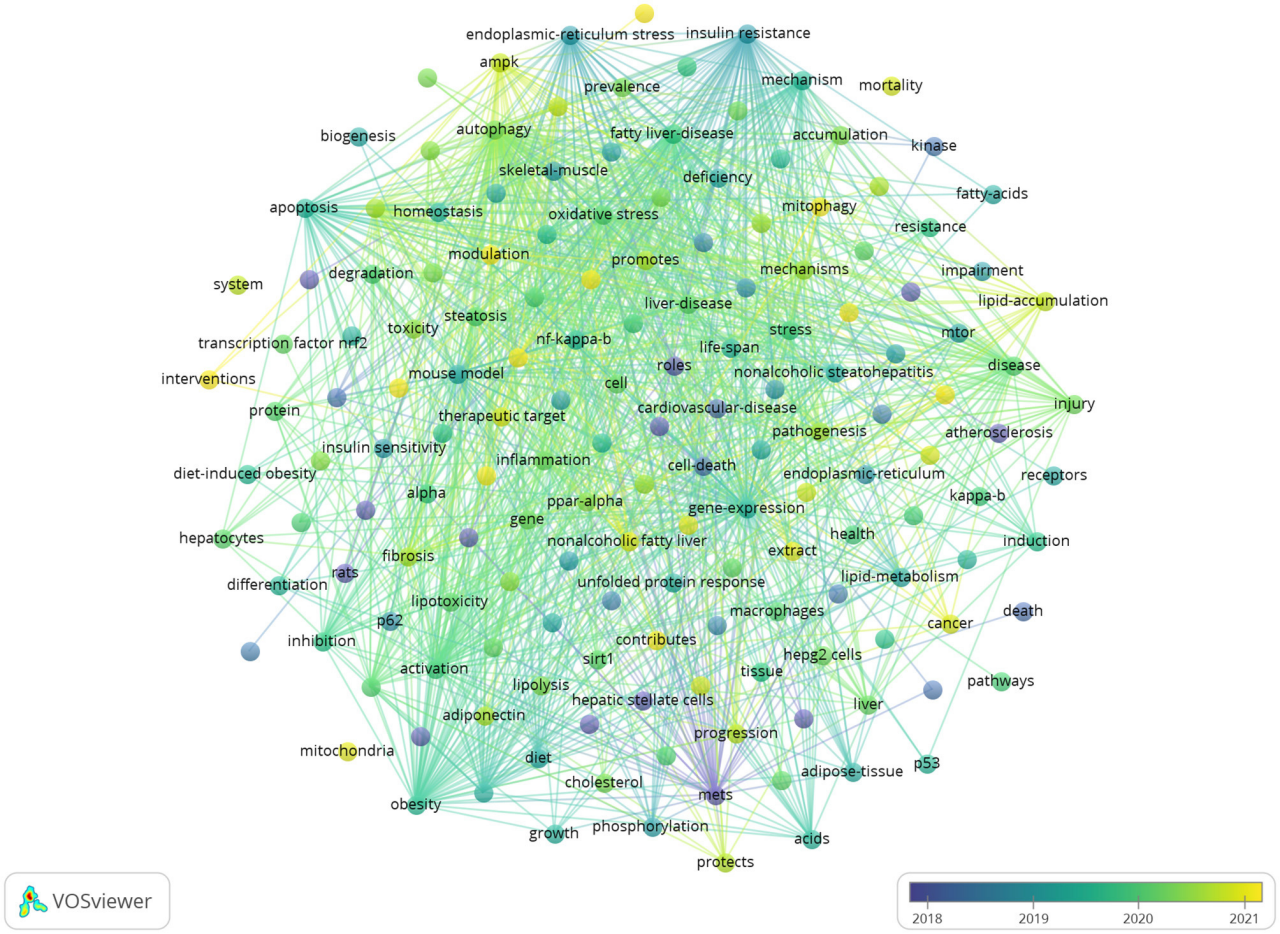
Visualization of keyword co-occurrence.

**Figure 9:**
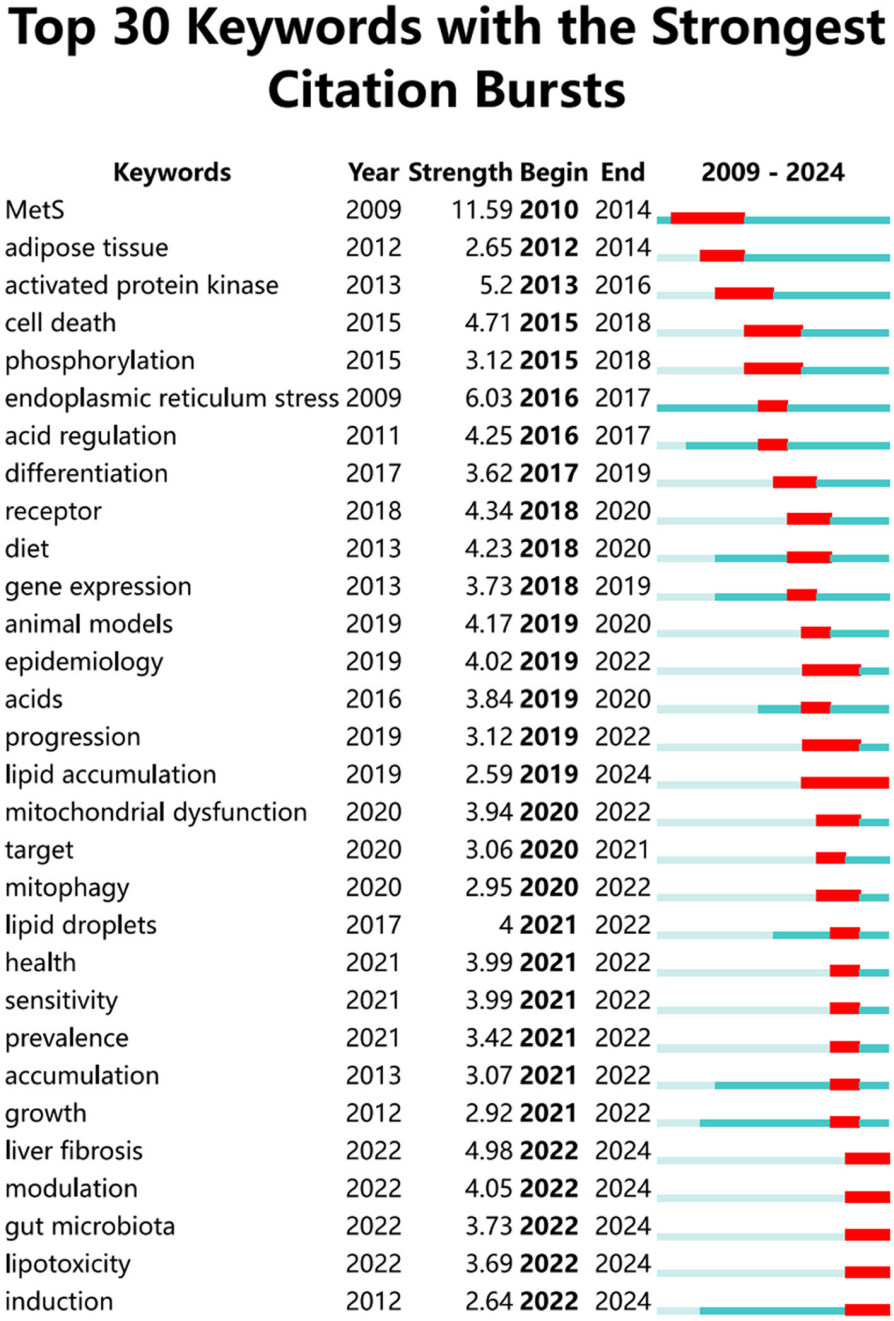
Top 30 keywords with the strongest citation bursts.

Keywords succinctly encapsulate the fundamental concepts of a paper, outlining the key areas of research interest. A comprehensive keyword analysis on the selected articles was performed using “Author Keywords” from the Biblioshiny application and “Keywords Plus” provided by the VOSviewer application. In total, 130 keywords with a minimum of 10 occurrences were identified in 2,337 keywords. However, upon comparing the results from these two sources, “Keywords Plus” was observed to provide more accurate results, making it the primary data source for the analysis. A network visualization map demonstrating the connections among these keyword co-occurrences was generated using VOSviewer. The sizes of the circles correspond to the frequency of occurrence of the keywords. A co-occurrence word analysis revealed that “insulin resistance,” “hepatic steatosis,” “oxidative stress,” “gene expression,” and “obesity” were the most frequently co-occurring keywords (Figure 8, Table 4). Figure 9 presents the top 30 keywords with the highest burst strengths. The most significant citation burst belongs to”MetS.” Particularly noteworthy is the concentration of keywords such as “Lipid accumulation,” “endoplasmic reticulum stress,” “liver fibrosis,” “modulation,” “gut microbiota,” “lipotoxicity,” “induction,” since 2022, indicating promising developments.

**Table 4:** Top 20 keyword co-occurrence network analysis.

**Id**	**Label**	**Occurrences**	**Total link strength**
685	Insulin resistance	144	526
570	Hepatic steatosis	72	284
946	Oxidative stress	51	205
494	Gene-expression	53	202
924	Obesity	43	167
52	Adipose-tissue	36	146
672	Inflammation	35	138
399	Endoplasmic-reticulum stress	31	128
867	Mouse model	35	126
1182	Steatohepatitis	29	121
752	Lipid-metabolism	26	93
578	Hepatocellular-carcinoma	25	91
355	Disease	21	83
34	Activation	19	75
820	metabolism	17	75
893	NF-kappa-b	16	73
804	Mechanisms	20	72
1108	Risk-factors	27	70
26	Acids	15	66
1183	Steatosis	16	60

## 4 Discussion

### 4.1 Overview publications analysis

Since 2009, studies on autophagy between MASLD and MetS have experienced rapid growth, particularly after 2014, driven by their pivotal biological roles in metabolic regulation mechanism research. It is evident that autophagy have gradually emerged as a hotspot in MASLD and MetS, indicated by an average citation of 32.01 per article. Additionally, the number of articles on autophagy in MASLD and MetS has steadily increased, with an annual growth rate of 31.60%.

### 4.2 Country, institution, journal, literature analysis

The countries with the highest publication volumes are primarily China, the USA, and Korea. China ranks first in total publications, while the USA and Canada exhibit the highest average citation rates, all exceeding 50. France demonstrates the greatest intermediary centrality, underscoring its active and prominent role in this field. Despite China possessing the highest total citations, it faces a relatively low average citation frequency of 23.5, suggesting a need for enhanced research quality, influence, and academic exchange among Chinese authors, thereby emphasizing the importance of publishing high-quality research. Notably, the three leading institutions contributing to publication volume are the Egyptian Knowledge Bank, Huazhong University of Science and Technology, and Yonsei University, indicating their pioneering roles in advancing autophagy-related research in MASLD and MetS. Albert Einstein College of Medicine and CNRS-National Institute for Biology (INSB) exhibit the highest intermediary centrality, serving as crucial contributors to fundamental autophagy research within these areas. The three most cited articles received 2,987, 733, and 729 citations, respectively, and were published in Nature (IF: 50.5), Comprehensive Physiology (IF: 4.9), and Nature Cell Biology (IF: 17.3) (13, 34, 35). All three articles focused on the mechanisms by which autophagy reg-ulates MASLD, highlighting the critical need for a mechanistic analysis of this metabolic dis-ease.

### 4.3 Key words time series analysis

Between 2009 and 2020, research keywords associated with autophagy in MASLD and MetS predominantly focused on terms such as “adipose tissue,” “activated protein kinase,” “cell death,” “phosphorylation,” “endoplasmic reticulum stress,” “acid regulation,” “receptor,” “differentiation,” “gene expression,” “animal models,” “epidemiology,” “progression,” and “lipid accumulation.” This trend signifies that comprehensive investigations have been con-ducted from multiple perspectives, incorporating intracellular mechanisms—such as signal transduction and cellular fate—as well three various research methodologies, including animal models, population studies, and the regulation of gene expression.

Since 2020, research on keywords related to autophagy in MASLD and MetS has in-creasingly centered on terms such as “health,” “mitochondrial dysfunction,” “target,” “mitophagy,” “lipid droplets,” “sensitivity,” “prevalence,” “accumulation,” “growth,” “liver fibrosis,” “modulation,” “gut microbiota,” “lipotoxicity,” and “induction.” This shift indicates a deeper exploration of cellular and subcellular structures, disease progression, autophagy regulation and intervention, as well as the relationship between gut microbiota and autophagy, including its induction. Moreover, keywords such as “lipid accumulation,” “liver fibrosis,” “modulation,” “gut microbiota,” “lipotoxicity,” and “induction” continue to gain prominence through 2024, underscoring the critical public health need for a more profound understanding of the mechanisms of autophagy in MASLD and MetS.

From 2010 to 2019, the keywords pertinent to this research encompassed intracellular mechanisms, research methodologies, gene expression regulation, and foundational multidi-mensional explorations of disease manifestation. In contrast, from 2020 to 2024, the focus has shifted to more nuanced areas, including cellular architecture, disease progression, autophagy regulation, gut microbiota, and disease susceptibility, alongside an increased emphasis on clinical applications. These findings suggest that autophagy may be closely linked to the search for new therapeutic targets and treatments, such as halting disease progression through the regulation of autophagy or improving disease conditions by indirectly influencing autophagy through the modulation of gut flora. Consequently, based on this bibliometric analysis, studies of autophagy in MASLD and MetS are likely to continue enhancing our understanding of their roles in the development of metabolic diseases and their potential as therapeutic targets.

### 4.4 Summary of autophagy in MASLD and MetS

MASLD encompasses a spectrum of liver disorders, ranging from steatosis to fibrosis and hepatocellular carcinoma. The pathogenesis of MASLD is primarily attributed to dysregulated lipid metabolism, oxidative stress, inflammation, and impaired autophagy (3, 8). Novel therapeutic approaches are being developed to restore autophagy and mitochondrial function while simultaneously addressing inflammatory and metabolic pathways. Natural products, including polyphenols [e.g., quercetin (15, 16), resveratrol (17, 18)], have been shown to promote AMPK-mediated autophagy and TFEB-driven lipophagy (19–21). Additionally, saponins [e.g., ginsenosides (36–39)] and terpenoids [e.g., andrographolide (40), cimigenol (41)] modulate SIRT1/AMPK signaling and enhance mitochondrial quality. Alkaloids [e.g., berberine (25, 42)] and flavonoids [e.g., naringin (43, 44), apigenin (45–47)] attenuate lipogenesis through AMPK activation and the modulation of endoplasmic reticulum (ER) stress. Moreover, additional agents such as probiotics (27), omega-3 polyunsaturated fatty acids (PUFAs) (48), and vitamin D (26) contribute to the amelioration of gut-liver axis dysregulation and oxidative stress. Key therapeutic targets identified in the literature include AMPK/mTOR, which serve as critical regulators of autophagy (18, 25, 49–54); TFEB, involved in lysosomal biogenesis (55, 56); the NLRP3 inflammasome, which is implicated in inflammatory processes (57); and PPARs, which play a pivotal role in lipid metabolism (58, 59). The gut hormone ghrelin enhances hepatic autophagy flux through the activation of AMPK signaling, thereby mitigating lipid accumulation and hepatocyte apoptosis in MASLD (14). This pathway presents a promising endogenous mechanism for the restoration of metabolic homeostasis. These findings highlight the potential of bioactive compounds that enhance autophagy and specifically target various pathways to mitigate the progression of MASLD (Table 5).

**Table 5:** Bioactive substances and therapeutic targets for NAFLD.

**Category**	**Bioactive substance**	**Author and year**	**Source (model)**	**Target pathway/protein**	**Mechanism**	**Ref**.
Flavonoids	Quercetin	Cao et al. (2023)	High-fat diet (HFD) rats	AMPK/mTOR	Activates AMPK-mediated mitochondrial autophagy	(15)
		Zhu et al. (2018)	HFD mice	IRE1α/XBP1s	Enhances VLDL assembly and lipophagy	(16)
	Naringenin	Yang et al. (2021)	HFD mice	AMPK	Enhances energy expenditure and autophagy	(44)
	Luteolin	Huang et al. (2023)	Palmitate-induced hepatocytes	ER stress/autophagy	Reduces lipotoxicity via ER stress modulation	(45)
Polyphenols	Resveratrol	Ding et al. (2017)	HFD rats	SIRT1	Alleviates ER stress via SIRT1 signaling	(17)
	Epigallocatechin-3-gallate	Zhou et al. (2014)	HepG2 cells	Autophagy markers	Stimulates hepatic autophagy and lipid clearance	(60)
Terpenoids	Formononetin	Wang et al. (2019)	NAFLD mice	TFEB	Promotes lysosome biogenesis and lipophagy	(19)
	Ginsenoside Rb2	Huang et al. (2017)	HepG2 cells	Sirt1/AMPK	Restores autophagy via Sirt1 activation	(36)
Alkaloids	Berberine	Singh et al. (2024)	HFD mice	SIRT1/AMPK	Activates autophagy and inhibits lipogenesis	(40)
Vitamins	Vitamin D	Abdelrahman et al. (2023)	NASH rats	NLRP3/autophagy	Modulates inflammasome-autophagy crosstalk	(61)
Probiotics	Probiotics	El-Din et al. (2021)	NAFLD rats	p-AKT/mTOR/LC3-II	Alleviates liver injury via autophagy	(27)
Saponins	Akebia saponin D	Gong et al. (2016)	Ob/ob mice	BNIP3	Induces mitophagy to alleviate steatosis	(62)
Others	Melatonin	Sun et al. (2023)	Ducks with Cd-induced NAFLD	PPAR-α	Restores autophagic flux via PPAR-α	(63)
	Taurine	Qiu et al. (2018)	Arsenic-induced NAFLD mice	Autophagic-inflammasomal	Inhibits pyroptosis via autophagy activation	(64)
Hormonal modulator	Ghrelin	Ezquerro S et al. (2019)	HepG2 cells	AMPK/mTOR	AMPK activation → autophagy enhancement	

### 4.5 Strengths and limitations

This bibliometric analysis represents the inaugural examination of the research status and trends concerning autophagy in MetS and MASLD over the past 15 years. This study offers scholars a comprehensive overview and stimulates new research inquiries, thereby fostering academic advancement within this domain. However, it is essential to acknowledge the limi-tations inherent in this study. Firstly, the analysis is restricted to English-language article sourced from the WoSCC database spanning the years 2009 to 2024, which may lead to potential gaps in the data available for analysis. Secondly, this investigation specifically retrieved publications associated with “autophagy,” MetS and MASLD, which may not capture the en-tirety of research conducted on autophagy in relation to MASLD and MetS. Nevertheless, the WoSCC database is recognized for housing some of the world's most influential and esteemed academic journals. The 1,114 articles analyzed in this study serve to mitigate these biases and provide an effective representation of the global status of research in this area.

## 5 Conclusion and outlook

This pioneering study provides a comprehensive analysis of the research trajectory and future prospects of autophagy in MASLD and MetS. There has been a significant increase in the number of publications in this area from 2009 to 2024. China has emerged as a leading contributor to this body of research, while France is recognized as the most influential country in the field. Enhanced global collaboration between China and France is essential to facilitate more extensive research. An analysis of journals indicates that autophagy research in MASLD and MetS constitutes a central focus within liver disease research, with prominent journals such as Scientific Reports and Hepatology playing a pivotal role in advancing the field. Keyword co-occurrence analysis reveals that the primary areas of focus include mitophagy, endoplasmic reticulum stress, insulin resistance, and other metabolic disorders. Notably, the interactions among mitophagy, lipid accumulation, oxidative stress, and inflammation are key topics of investigation. Current research on underlying mechanisms primarily revolves around the NF-kB and AMPK pathways, while cutting-edge studies are exploring avenues such as PI3K, NRF2, and mTOR. These findings provide timely insights that may inform therapeutic approaches for managing fatty liver disease and assist researchers in selecting appropriate journals for publi-cation, identifying potential collaborators, and remaining abreast of current trends and devel-opments in the field, thereby advancing overall knowledge.
